# IgTreeZ, A Toolkit for Immunoglobulin Gene Lineage Tree-Based Analysis, Reveals CDR3s Are Crucial for Selection Analysis

**DOI:** 10.3389/fimmu.2022.822834

**Published:** 2022-10-26

**Authors:** Hadas Neuman, Jessica Arrouasse, Meirav Kedmi, Andrea Cerutti, Giuliana Magri, Ramit Mehr

**Affiliations:** ^1^ The Mina and Everard Goodman Faculty of Life Sciences, Bar Ilan University, Ramat Gan, Israel; ^2^ Division of Hematology and Bone Marrow Transplantation, Chaim Sheba Medical Center, Ramat Gan, Israel; ^3^ Sackler School of Medicine, Tel-Aviv University, Tel Aviv, Israel; ^4^ Translational Clinical Research Program, Institut Hospital del Mar d’Investigacions Mèdiques (IMIM), Barcelona, Spain; ^5^ Catalan Institute for Research and Advanced Studies (ICREA), Barcelona, Spain

**Keywords:** antibody, B cell receptor (BCR), B cell repertoire, immunoglobulin, lineage tree, somatic hypermutation (SHM)

## Abstract

Somatic hypermutation (SHM) is an important diversification mechanism that plays a part in the creation of immune memory. Immunoglobulin (Ig) variable region gene lineage trees were used over the last four decades to model SHM and the selection mechanisms operating on B cell clones. We hereby present IgTreeZ (Immunoglobulin Tree analyZer), a python-based tool that analyses many aspects of Ig gene lineage trees and their repertoires. Using simulations, we show that IgTreeZ can be reliably used for mutation and selection analyses. We used IgTreeZ on empirical data, found evidence for different mutation patterns in different B cell subpopulations, and gained insights into antigen-driven selection in corona virus disease 19 (COVID-19) patients. Most importantly, we show that including the CDR3 regions in selection analyses – which is only possible if these analyses are lineage tree-based – is crucial for obtaining correct results. Overall, we present a comprehensive lineage tree analysis tool that can reveal new biological insights into B cell repertoire dynamics.

## Introduction

SHM can take place in germinal centers (GC) as well as in extrafollicular (EF) sites (1) and introduces base-pair changes into rearranged Ig variable region genes. B cells that gain mutations that improve their receptors’ affinity to the antigen are selected to expand, and eventually generate plasma cells and memory B cells with an improved receptor affinity (2). The resulting high-affinity memory B cells and long-lived plasma cells allow faster and more efficient secondary immune responses. Therefore, SHM is an important mechanism in the generation of broad and effective immune responses.

**Figure 1:**
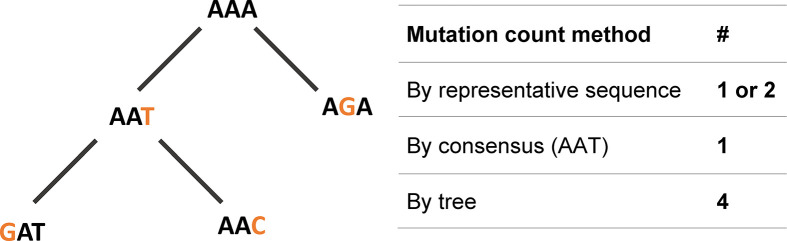
Mutations are best described, and more precisely counted, using lineage trees. In the example one-codon “clone”, a mutation count that is based on a representative sequence results in one or two mutations. Using a consensus sequence, which is AAT for this clone, results in 1 mutation. Only a tree-based mutations count results in the exact number of mutations, which in this case is 4.

The above-described affinity maturation can be modeled using Ig gene lineage trees. A lineage tree (sometimes also called pedigree or dendrogram) is a rooted tree, similar to a phylogenetic tree, in which nodes correspond to B cell receptor chain variable region gene sequences. The shapes of lineage trees hold information on the dynamics of the GC response that generated the trees (3). Properties such as the degree of branching can point to the strength of selection and initial affinity to the antigen. Insights on transitions between populations, such as class switch recombination (CSR), cell differentiation and migration, can be deduced from the relationships between tree nodes. Finally and most importantly, tree-based mutation analysis is more accurate than analysis based on comparing each sequence to the putative germline sequence (**Figure 1**) (4, 5).

Over the last three decades, lineage trees were used to elucidate many features of the B cell repertoire dynamics. Meng et al. created an atlas of the B cell distribution and found that the repertoire is divided into two major networks of large clones, one in the blood, bone marrow, spleen and lung, and another in the gastrointestinal track (6). Tipton et al. found in 2015 that activated naïve B cells are the precursors for antibody-secreting cells in Systemic Lupus Erythematosus (7). Tabibian-Keissar and colleagues found evidence for B-cell trafficking between gut-lymph node in ulcerative colitis (8) and Hoehn et al. used phylogenetic model to characterize the effects of aging on B cell repertoire development and B cell responses to influenza vaccination (5). Overall, the analysis of lineage tree properties sheds light on affinity maturation and the diversification of Ig genes in health and in various pathological conditions. However, these analyses were done using different, separate tools, each of which reveals different aspects of lineage trees.

Diffuse large B-cell lymphoma (DLBCL) is the most common form of lymphoma and accounts for 25–35% of all non-Hodgkin lymphomas. About 30–50% of patients treated with the standard-of-care therapy are either refractory to treatment or have relapsed disease after the complete response (9). Detecting an early stage of a relapsed disease may have a positive impact on the therapy outcome. There is a wide interest in applying machine learning on biological fields and more precisely on Adaptive Immune Receptor Repertoire (AIRR) data sequencing, from an adaptation of the natural language processing (NLP) technique for B cell receptor (BCR) sequencing data (10) to disease classification based on BCR repertoires (11). Here, we demonstrate that we can apply machine learning classification models to the IgTreeZ (mutation analysis) output, and use it to distinguish between lineage trees from DLBCL patients and those from healthy controls.

This study presents a comprehensive python-based tool for the analysis of many aspects of Ig gene lineage trees. The program was developed on LINUX and most of its features are compatible with Windows as well (including the population and topology analyses, filtering and drawing). The next versions will be fully compatible with all operating systems. IgTreeZ allows the analysis of population transitions, tree topology, and mutations at the repertoire level. The program also includes utility scripts for filtering trees by population and size, and for graphical and statistical comparisons of the results of analyzing more than one repertoire. Finally, we used simulated and empirical data to demonstrate the usefulness of this tool and the potential of lineage tree-based Ig gene repertoire analysis, and applied a successful classification model on IgTreeZ’s mutation counts of DLBCL patients and healthy controls.

## Methods

### Mutation Analyses

An Ig gene lineage tree represents the diversification of a B-cell clone. Hence, a mutation count based on tree topology is more accurate than counting the mutations on each sequence separately (4, 5). Currently in IgTreeZ, prior to mutation count, the nodes are linked to the corresponding sequences. Sequences can be given as a Fasta file or as AIRR/Change-O database, or as an AIRR JSON rearrangement scheme, which includes the trees and sequences. Once given, each of the sequences is linked to the corresponding node by name. Hypothetical nodes, created by the tree inference program, sometimes lack representation in the input files. In the case of an internal node with no corresponding sequence, if the edge between the node and its parent indicates a zero distance – the node is linked to its parent’s sequence; if not, a consensus sequence is generated, with a priority to gaps and Ns (that is, if the number of Ns is equal to the number As in a certain position, the program prefers the N, or a gap, over the A), to avoid mutation over-count.

Once all nodes on the tree are linked to their sequences, the program traverses all tree nodes, counts all the observed mutations, and characterizes each mutation by its location (CDR/FWR), based on IMGT region definitions (12). Since each tree is handled separately, mutations in the CDR3 can also be identified as CDR mutations; the program compares CDR3 nucleotides to the clone’s consensus CDR3, and thus counts only mutations generated by SHM and not the diversity generated by N/P nucleotide. The program also characterizes each mutation by its nucleotide source, mutation type (transition/transversion) and amino-acid change (replacement/silent). If it is a replacement mutation, the program characterizes also the amino-acid type – charge, hydropathy, volume, chemical, hydrogen donor or acceptor atoms and polarity, based on the IMGT physicochemical amino acid classes (13, 14). The resulting data are saved as a CSV file with mutation counts for each tree, and graphs can be included in the output using the ‘plot’ argument.

### Selection Analyses

Selection analysis is based on IgTreeZ’ mutation analysis, which calculates the number of silent and replacement mutations in the CDRs and FWRs for all sequences each tree. The resulting counts are sent, together with the corresponding germline sequence and the length of the CDR3 of each tree, to ShazaM (15, 16). Using ShazaM, we calculate the expected mutation frequency, estimate the selection strength for each tree, and compare the selection scores of multiple repertoires. We include the CDR3 by modifying the region definition parameter according to each tree’s CDR3 length and calculating the expected mutation frequency for each germline separately. All these steps were performed by our R script ‘shazam_selection_on_igtreez_output.r’, which is included in IgTreeZ.

### Tree Topology Analysis (MTree)

This analysis quantifies the shape properties of Ig gene lineage trees. This was first suggested by Shannon and Mehr (17), who postulated that lineage tree shapes can be used to reveal the dynamics of hypermutation and antigen-driven selection in GCs. Later, seven variables were found to have a significant correlation with several B cell response parameters (3). Recently, we wrote a python version of MTree^©^, which calculates these seven variables for each tree in parallel and saves them in CSV files. The MTree results of different repertoires are compared using a second tool, which creates a box plot for each variable, and a scatter plot of each pair of variables. IgTreeZ now includes this analysis as part of its functions.

### Tree Drawing

The tree drawing function of IgTreeZ aims to visually illustrate lineage tree shapes. We base our drawing on the graph description language DOT, as implemented in the Graphviz program (18). The input Newick-format trees are first translated to the DOT language. The translated DOT files can then be saved as image files (in several formats) or colored for quick impression. The coloring is based on the cell population names associated with tree nodes, with multi-population nodes colored with multiple colors. The font size and line width can also be adjusted using program parameters. Examples are given in the *Results* section.

### Tree Filtering

Some comparative analyses require choosing only trees with certain characteristics, such as trees belonging to specific cell populations or tissues. To address this need, we developed a script to filter trees based on tree size (number of nodes or leaves), population composition or other features. Filtering by features such as population composition is done using one of three logical gates: AND, which selects trees composed of nodes associated with all the given populations; OR, which select trees composed of nodes associated with at least one of the given populations; and NOT, which selects trees that lack nodes associated with any of the given populations. The selected tree names are saved in a CSV file, and these trees can be automatically copied to a new directory using a parameter.

### Simulation

To test our mutation counts in a CDR/FWR region connotation, we used Yermanos et al.’s AbSim simulation (19). We simulated 100 lineages under each condition, using the ‘data’ SHM method, which focuses mutation events during SHM to the CDR regions (defined based on IMGT), and has an increased probability for transition mutations relative to transversions. We used different probabilities for SHM nucleotide changes, different probabilities for a given sequence to undergo SHM, and different baseline probabilities for each nucleotide to be mutated. We annotated the output sequences using IgBlast version 1.14.0 (20) on IMGT/GENE-DB (21) reference sequences from March 26, 2020, and built parsimony trees using AlakazaM version 1.0.1 (16). Overall, more than 2800 trees were simulated and analyzed.

### Empirical Data Processing

#### Healthy Ileum Data

We used Ig sequencing data from sorted B cell subsets from histologically normal human ileum tissue samples of two adult individuals (**Table 1**), which were barcoded with unique molecular identifiers, amplified, sequenced and analyzed using our lab’s pipeline (Tejedor Vaquero S et al. manuscript in preparation). Briefly, the sequences were preprocessed using pRESTO version 0.5.8 (22), annotated online using IMGT/HighV-QUEST version 3.4.15 (23) with the IMGT/GENE-DB (21) reference sequences from January 17, 2019, and processed and assigned for clones using Change-O version 0.4.1 (16). Lineage tree construction was performed using IgTree^©^ (24).

**Table 1:** Cerutti Lab dataset – patient information.

Patient number	Gender	Age	B cell subsets
**3**	Female	35	Naïve, Memory, GC, Plasma cells
**4**	Female	63	Naïve, Memory, GC, Plasma cells

#### COVID-19 Data

We used data obtained from Montague et al. (25), who sequenced and analyzed B cells from blood samples of COVID patients in various severity levels at several time points from the appearance of clinical symptoms. We downloaded sequences of 20 samples (including replicates) from three patients with different disease severity from different time points, and from three healthy controls (**Table 2**). All sequences were downloaded from iReceptor (26). We annotated the sequences using IgBlast version 1.14.0 (20) on IMGT/GENE-DB (21) reference sequences from March 26, 2020. We filtered the functional sequences and assigned them into clones, based on trimming thresholds, using Change-O (16); and built parsimony trees using AlakazaM version 1.0.1 (16). Trees were not constructed for clones with more than 3000 sequences, for the sake of rapid analysis.

**Table 2:** COVID-19 donor data.

Severity	Subject ID	Sex	Age	Collection time (days from symptom onset)
**Healthy**	H1	F	28	
**Healthy**	H2	F	30	
**Healthy**	H3	M	45	
**Mild**	2	F	37	2
**Mild**				15
**Mild**				34
**Moderate**	8	M	37	14
**Moderate**				32
**Severe**				34
**Severe**	18	F	62	8
**Severe**				30

#### Data for Machine Learning Models

We used data from three datasets. Data of one healthy donor were obtained from (27) and downloaded from iReceptor (26), and another healthy donor’s data were obtained from (28) directly. Six peripheral blood and bone marrow samples of four DLBCL patients were sequenced by Kedmi et al. (**Table 3**). All the DLBCL samples were taken from relapsed patients before treatment. Data were analyzed and lineage trees generated as described in section 2.7.2.

**Table 3:** Healthy control and DLBCL patient datasets.

Group	Sample name	Patient	Tissue	Study	# Trees
**Healthy Controls**	H1	H1	PB	(27)	18,068
	H2	H2	PB	(28)	9,190
	Overall healthy control trees				27,258
**DLBCL**	D1	P1	PB	Kedmi et al.	1,651
	D2	P2	PB		1,309
	D3	P3	PB		35
	D4	P4	BM		738
	D5	P1	BM		2,419
	D6	P2	BM		4,834
	Overall DLCBL trees				10,915

### Machine Learning Classification Model Application to IgTreeZ Mutation Count

#### Tree-Based Mutation Analysis and Dataset Preprocessing

Mutations on the DLBCL and healthy control lineage trees were analyzed using IgTreeZ. We defined the resulting mutation counts as the features for the machine-learning models, together with one additional feature that represents the number of mutations per sequence (**Supplementary Table 1**). A binary column named ‘status’ with the value of 1 for patients and 0 for controls was added. The control and patient data frames were concatenated. All “Nan” values were replaced with 0 (since Nan values mean no mutations were found).

#### Data Resampling

We chose to resample the training set because our data were imbalanced (**Table 3**), and over-sampling of the DLBCL data caused overfitting. We tried a combination of over-and under-sampling (over-sampling the patient data, and under-sampling the healthy control data) using methods such as SMOTETomek, which combines SMOTE and Tomek links. However, this also caused overfitting, together with low prediction scores. Using a specific pipeline to choose the required sample size also caused overfitting together with low predictions scores. We concluded that any over-sampling causes overfitting. Therefore, we chose to under-sample the control data using OneSidedSelection (since a random under-sampling also returned low prediction scores), and only under-sampled the training set and not the test set, to make the test as reliable as possible.

#### Machine Learning Models

Six machine learning models were built (using the scikit-learn package in python) to predict whether a tree-based mutation count originated from a healthy person or a DLBCL patient. The feature values (except the first two columns – sample name and tree ID) were defined as the input, and the status as the output. We chose to scale our data using Normalizer, after trying 8 different scaling methods and finding that Normalizer returns the best results. This can be due to the Normalizer scaling method using rows while other scalers use columns. Since each row in our data represents a separate tree, using Normalizer made the most sense. Data were split into training (75%) and testing (25%) set. To optimize the hyperparameters of all the models, we used GridSearchCV, a method for hyperparameter tuning in which we define a grid of possible parameter values (**Supplementary Table 2**), and GridSearch searches for the optimal set of hyperparameter combinations, using the k-fold cross-validation (CV) approach (cv = 5). In other words, this method trains the model using different combinations of the above-mentioned features and gives the best combinations based on the optimal k-fold CV score obtained.

## Results

### Tree-Based Selection Analysis Reflects Mutation Distributions and Shows Inclusion of CDR3s Is Crucial

A lineage tree-based mutation analysis is obviously more accurate than an analysis based on comparing each sequence to the germline (4, 5). On trees, the ancestor sequence for each mutation is better defined; moreover, successive mutations on the same nucleotide, including reversion mutations, can be identified (**Figure 1**). In addition to a more accurate mutation identification and counts on the V and J segments, a tree-based analysis makes it possible to account for mutation in the CDR3 region, which is better defined for a clone (where the putative “germline” CDR3 sequence is clone’s consensus CDR3 sequence, and the tree tracks mutations on the CDR3 as well as the V and J segments) than for a single sequence (where the putative “germline” CDR3 sequence is identical to that of the sequence, and hence no mutations can be identified there). Since selection analysis is based on mutation counts, this analysis can also benefit from the more precise lineage tree-based mutation counts.

**Figure 2:**
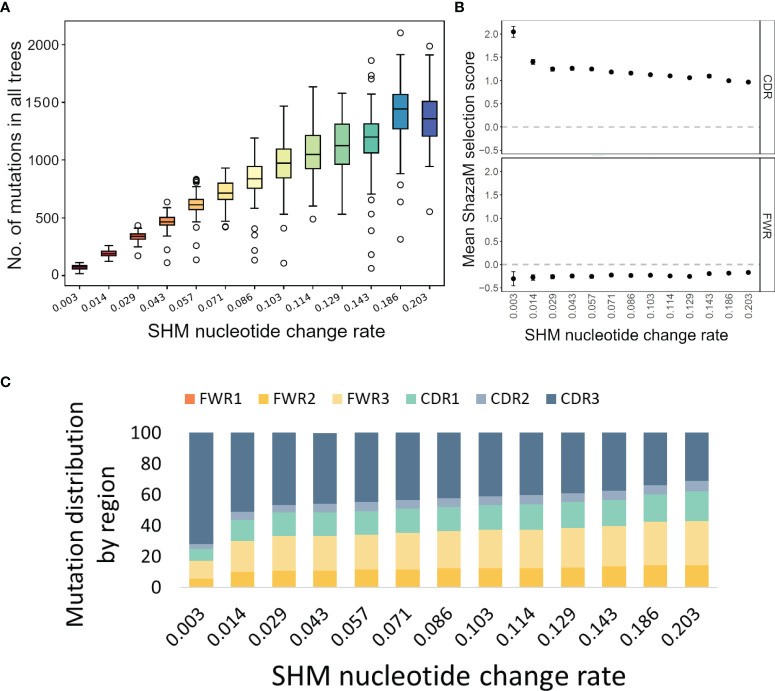
Mutation and selection analyses of simulated trees. **(A)** Mutation counts per whole sequence based on simulated trees under different probabilities for SHM nucleotide change. The plot was created using the compare-reps script based on IgTreeZ-mutations result. **(B)** The means and confidence intervals of the selection scores for tree-based mutation count as calculated and plotted using ShazaM (15, 16). **(C)** The distribution of mutations in the different regions, as counted by IgTreeZ’ program poptree-mutations. All trees and sequences were simulated using AbSim (19). CDR, complementarity determining region. FWR, framework region.

To test these functions of IgTreeZ, we used Yermanos et al.’s AbSim simulation, which is a time-resolved antibody repertoire simulation that enables the modeling of several immunologically relevant parameters (19). We tested both our tree-based mutation count, especially in the CDR/FWR regions, and its influence on the selection analysis. We modified three simulation parameters and found that the SHM nucleotide change rate has the most impact on mutation profiles. We simulated 100 lineages for each of 13 different probabilities for SHM nucleotide change. By analyzing the resulting 1300 trees we found that, as expected, more mutations are counted, in both the CDRs and FWRs, with higher SHM nucleotide change rate (**Figure 2A**). However, when we used these counts to estimate the selection strengths using ShazaM (15, 16), we found opposite trends – the selection for replacement mutations is weaker as the SHM nucleotide change rate is higher in the CDR. The FWR presents stable selection against replacement mutations (**Figure 2B**). To understand the reason for these opposite trends in the CDRs, we tested the mutation distribution in each region and found that, indeed, the mutation counts in the CDR3 region become relatively greater than those of other regions as the SHM nucleotide change rate is decreased (**Figure 2C**). These results emphasize the importance of the inclusion of the CDR3 in the selection analysis, and thus the potential of lineage tree-based selection analysis, which enables the inclusion of the CDR3 region.

### Different Mutation and Selection Patterns in B-Cell Sub-Populations in Human Gut

**Figure 3:**
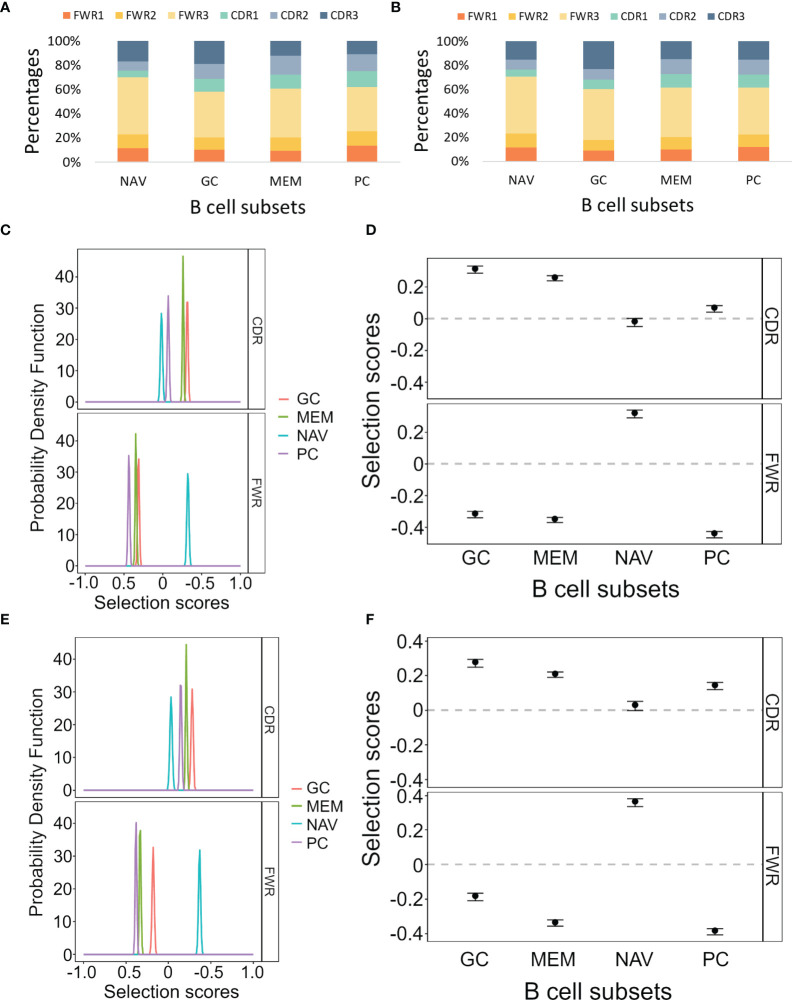
Mutation and selection analyses of IgV gene sequences from different ileal B cell and plasma cell subsets. **(A, B)** The mutation distributions as counted by the IgTreeZ program function “mutations” for Donor 3 and Donor 4, respectively. **(C, E)** The probability density function of the selection scores for the lineage tree-based mutation counts of the different cell subsets for Donor 3 and Donor 4, respectively. **(D, F)** Means and confidence intervals of the selection scores for the same mutation counts for Donor 3 and Donor 4, respectively. Panels **(A–D)** were calculated and plotted using ShazaM (15, 16). MEM, memory; PC, plasma cell; NAV, naïve; CDR, complementarity determining region; FWR, framework region.

In order to test our program on empirical data, we analyzed lineage trees from sorted B cells from two histologically normal ileal tissue samples (**Table 1**) processed using our lab’s pipeline (Tejedor Vaquero S et al. manuscript in preparation). We used IgTreeZ to record the mutation distributions in these trees and found several consistent patterns, as follows. The relative fractions of CDR3 mutations were highest in GC cells (18 and 23% in Donor 3 and 4 respectively, **Figures 3A, B** and **Tables 4**, **5**). The relative fractions of FWR3 mutations were highest in naïve cells, but not those of the FWR1 and FWR2 regions. Correspondingly, the overall CDR region mutation relative fractions were lowest in naïve cells. Plasma cells of both donors exhibited the highest relative fractions of mutations in FWR1 regions (13 and 12% in donor 3 and 4, respectively), and the lowest relative fractions in FWR3 regions (36 and 39% in donor 3 and 4, respectively).

**Table 4:** Donor 3 mutation distribution.

Region	Naïve (%)	GC (%)	Memory (%)	Plasma (%)
**FWR1**	11.6	10.2	9.3	13.4
**FWR2**	11.4	10	10.9	12
**FWR3**	46.7	37.9	40.5	36.2
**CDR1**	5.6	10.4	11.5	13.1
**CDR2**	7.7	12.6	15.4	14.1
**CDR3**	17	18.9	12.4	11.1

**Table 5:** Donor 4 mutation distribution.

Region	Naïve (%)	GC (%)	Memory (%)	Plasma (%)
**FWR1**	11.3	9	9.8	12
**FWR2**	12	8.5	10.7	10.2
**FWR3**	47	42.6	41.6	39.1
**CDR1**	5.8	7.7	11.1	11
**CDR2**	8.4	9	12.6	12.6
**CDR3**	15.5	23.2	15.2	15.2

Using the ShazaM R package (15, 16) to perform selection analysis on the IgTreeZ mutation counts revealed consistent results in both donors, in which GC cell lineage trees exhibited the strongest selection for replacement mutations in the CDRs, and plasma cell lineage trees show the strongest selection against replacement mutations in the FWRs (**Figures 3C–F**). This may suggest that gut plasma cells are selected for their receptors’ stability, rather than affinity. Memory B cell lineage trees exhibited a slightly weaker selection for replacement mutations than GC cell lineage trees in the CDRs, and naïve cell lineage trees seem to undergo the weakest selection in CDRs and strong selection for replacement mutations in FWRs. These findings illustrate the different mutation and selection courses of each B cell sub-population in human gut, and, again, the potential of lineage tree-based mutation and selection analyses.

### Lineage Tree-Based Transition, Mutation and Selection Analyses of Data From COVID-19 Patients Elucidates the Immune Response to SARS-COV-2

**Figure 4:**
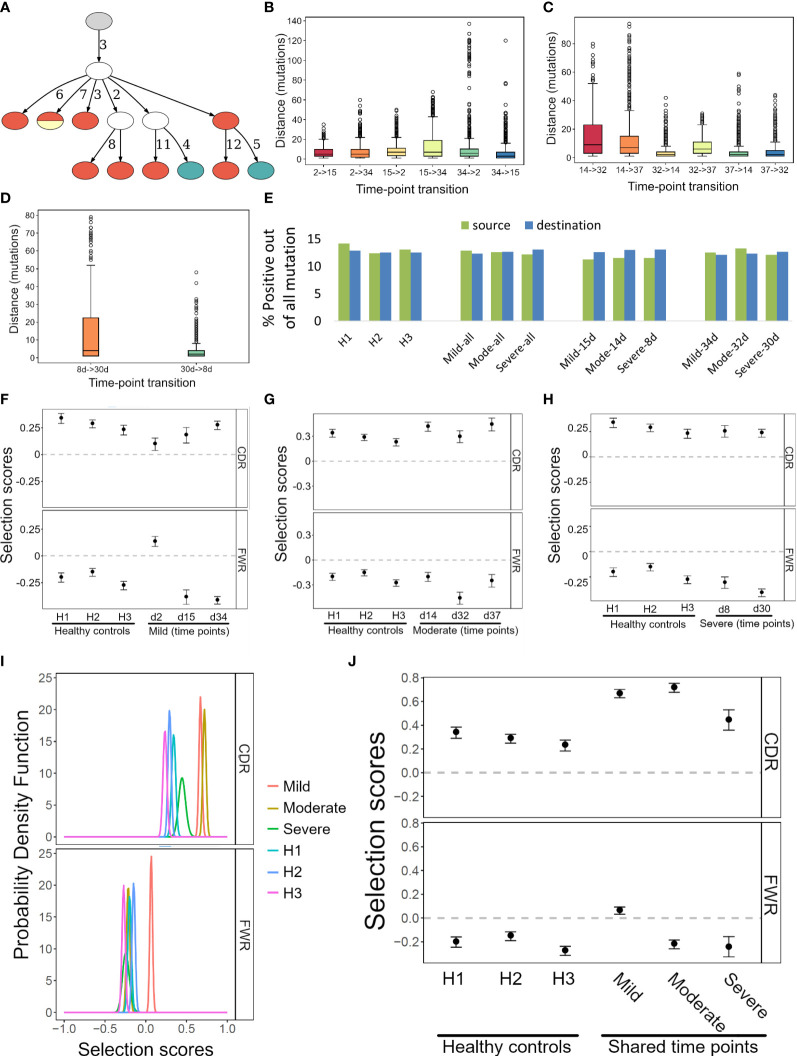
Transition, mutation and selection analyses of human B cells of COVID-19 patients and healthy donors. **(A)** An example lineage tree from the patient with mild disease. Filled nodes represent sampled sequences, nodes with more than one color represent multiple sequences from different time points, and the numbers on edges correspond to numbers of mutations between nodes. The graph was created using the function IgTreeZ-draw. **(B–D)** The numbers of mutations involved in transitions for the mild, moderate, and severe disease patients. The plots were created using IgTreeZ-poptree. **(E)** The partial fractions of the mutations that involved positive amino acids out of all mutations. Source/Destination – the mutation source/destination that involved a positive amino acid. **(F–H)** The mean and confidence interval of the selection scores for the tree-based mutation counts of the mild, moderate, and severe disease patients. **(I)** The probability density function of the selection scores for clones that were shared between time points of the mild, moderate, and severe disease patients and of healthy donors. **(J)** The mean and confidence interval of the selection scores for the tree-based mutation counts of the same data as I. Panels **(D–F, I, J)** were calculated and plotted using ShazaM (15, 16).

To further demonstrate the potential of IgTreeZ, we analyzed B cell sequences from three COVID-19 patients that differed in disease severity, obtained by Montague et al. at two or three time points from clinical symptom onset for each patient (**Figure 4A**), and of three healthy donors (**Table 2**) (25). We annotated the sequences, labeled the sequences by time point, and constructed lineage trees using AlakazaM (16). Next, we used IgTreeZ to filter trees based on their time point composition, to analyze the transitions between time points, and to perform tree-based mutation and selection analyses.

Counting the number of mutations involved in transitions between time points (defined as transition distance) reveals that transitions between day ~15 to day ~30 and between day ~8 to day ~30 included the highest mutation counts (**Figures 4B–D**). This indicates an extensive GC response that took place at these times. Of note, impossible transitions (such as from day 30 to day 8) were also found, as tree construction algorithm are geared towards choosing the minimal tree out of the infinite number of possible trees, and the minimal tree is not always the best representation of the actual response; the same mutation could, in reality, occur simultaneously in parallel branches of the clone. However, the transition distances associated with impossible transitions were low (median of 2 mutations in the patients with severe and moderate disease, and 3-7 mutations in the patient with mild disease). This implies that the impossible transitions were probably the results of incorrect inference of the relationships of very similar sequences, as a result of the requirements of tree generating algorithms. It is not recommended to constrain such algorithms, because this will result in trees that are more grossly incorrect.

IgTreeZ’ mutation analysis function includes mutation profiling, in which mutations are characterized by their source and destination nucleotides, their location (FWR or CDR), and their type (transition or transversion, silent or replacement mutation). In addition, replacement mutations are characterized by source and destination amino acid charge, hydropathy, volume, chemical, hydrogen donor or acceptor atoms and polarity. We analyzed the data from COVID-19 patients and healthy donors using this function, and compared the results by subject and time point. The relative fractions of mutations involving a positive amino acid out of all the source mutations was the lowest in COVID-19 patients at time points 8-15 days. Moreover, the relative fractions of mutations involving a positive amino acid out of all the destination mutations in COVID-19 at these time points was among the highest (**Figure 4E**). This suggests a tendency of the affinity maturation in COVID-19 patients to avoid mutating positively charged amino acids, and to favor mutation that create such amino acids.

Next, we used ShazaM to evaluate selection strengths in the same lineage tree repertoires. The three patients’ lineage trees showed the strongest selection against replacement mutations in FWRs around 30 days from clinical symptom onset (**Figures 4F–H**). This indicates dominance of affinity-matured, structurally stable antibodies. On the other hand, the selection for replacement mutations in CDRs showed dynamic changes in the mild and moderate disease patients, but not in the severe disease patient. This may reflect the absence of an effective antibody selection process in the latter patient. On day 2, the mild disease patient showed the weakest selection for both the CDRs and the FWRs. This may indicate the generation of a large number of low affinity, pre-GC BCRs. Finally, to focus our analysis on clones that may have been involved in the immune response to SARS-COV-2, we filtered clones that were sampled at all three time points. Such clones had higher selection for replacement mutations in the CDRs (**Figures 4I, J**). Moreover, the patient with the severe COVID-19 disease showed the lowest selection for replacement mutations among the three patients.

### Machine Learning Models Using IgTreeZ Output Can Distinguish Lymphoma From Normal Lineage Trees

IgTreeZ mutations analysis returns an extensive data table. This data can be used for machine learning. To demonstrate the potential of this application, we built six machine learning models to predict whether a tree-based mutation count originated from a healthy person or a DLBCL patient. Preceding model construction, we performed an exploratory data analysis (**Supplementary Figures S1**, **S2**) and a dimensionality reduction. We used PCA with two and three dimensions, and T-SNE, and found that all of them separated the data very well (**Supplementary Figure S3**). Therefore, we used the full dataset for the different models. Among the six models tested, the Support-Vector Machine (SVM) returned the best results.

#### SVM Model

**Figure 5:**
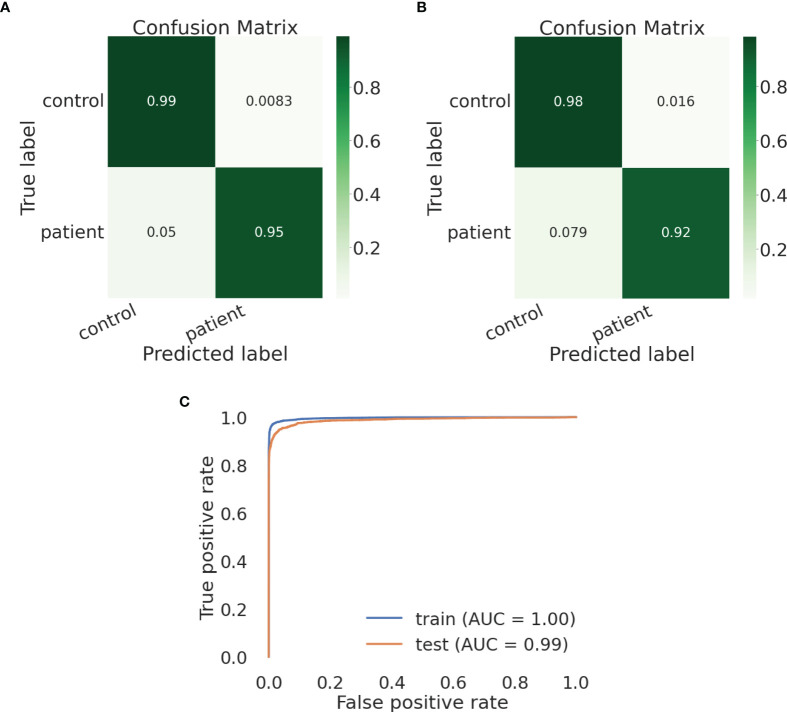
An SVM model can distinguish lymphoma from healthy control lineage trees using IgTreeZ output. **(A, B)** Confusion matrices for the training and test set, respectively. **(C)** Roc curves for both sets. Confusion matrices and ROC curves were created using Python’s scikit-learn package.

We built an SVM model and tested a parameter space to obtain the optimal parameter values. We chose to define the following parameter options: kernel (the kernel type to be used in the algorithm) - [‘poly’, ‘rbf’, ‘sigmoid’] and C (the regularization parameter) - [50, 10, 1.0, 0.1, 0.01]). We defined gamma, the kernel coefficient, to be ‘scale’. We found the optimal parameter values to be kernel=rbf and C=10. Using these parameter values, the training and test sets show similar and high results – the macro avg scores are 0.97085 for the training and 0.95784 for the test sets, respectively (**Tables 6**, **7**, respectively). The confusion matrices are also consistent, and the true prediction rate of the control trees was 0.99 for the training set and 0.98 for the test set, while the true prediction rate of the DLBCL trees was 0.95 and 0.92 for the training and test sets, respectively (**Figures 5A, B**, respectively). The ROC was 1 and 0.99 for the training and test sets, respectively, and overall exhibits a very high learning rate (**Figure 5C**).

**Table 6:** Classification report of the training set.

	Precision	Recall	f1-score	support
**0**	0.95235	0.99111	0.97134	8407
**1**	0.99079	0.95074	0.97036	8263
**accuracy**			0.97086	16470
**Macro avg**	0.97157	0.97092	0.97085	16470
**Weighted avg**	0.97164	0.97086	0.97085	16470

**Table 7:** Classification report of the test set.

	Precision	Recall	f1-score	support
**0**	0.96985	0.98304	0.97640	6838
**1**	0.95589	0.92325	0.93929	2723
**accuracy**			0.96601	9561
**Macro avg**	0.96287	0.95314	0.95784	9561
**Weighted avg**	0.96587	0.96601	0.96583	9561

#### KNN, Decision Trees, Random Forest, AdaBoost and LDA Models

We chose to define parameter options for KNN, decision trees, Random Forest, AdaBoost and LDA models as shown in **Supplementary Table 2**. LDA results exhibit a small increase from the training set to the test set. However, since we analyzed a relatively small dataset, this increase is negligible. Since Random Forest and AdaBoost returns information on feature importance, we re-trained a simple, non-cross validation model, with the optimized parameters we found earlier. We found that the two most important features for Random Forest are ‘sequences’ and ‘nodes’ and for AdaBoost these are ‘sequences’ and ‘mutations_per_sequence’ (**Supplementary Tables 3**, **4** for Random Forest and AdaBoost, respectively), and tested their distributions (**Supplementary Figure S4**). From the ‘mutations_per_sequence’ distribution, it is apparent that DLBCL trees tend to have fewer mutations per sequence, with a narrower distribution than the control trees. On the other hand, we can see that the DLBCL trees have more diverse sequence and node numbers, and tend to have more sequences than the control trees. The two features seem to be two sides of the same coin – the DLBCL clones are larger, but this is mostly due to branching, as they contain fewer unique mutations in each sequence.

## Discussion

In this work, we present IgTreeZ, a comprehensive tool for lineage tree analysis, and demonstrate the value of this tool. Several studies emphasized the importance of lineage tree-based mutation and selection analysis. Zuckerman et al. predicted an alteration in the SHM mechanism in myasthenia gravis thymic ectopic GCs that was later verified by gene expression analysis (4). Yaari and colleagues used lineage trees to show that the FWR and CDR are designed under different selection patterns and that long-term selection is dependent on the heavy chain variable gene family (29). Lineage tree-based mutation counts can reveal multiple mutation patterns that are under-counted using clone consensus sequences and over-counted using a direct sequence-based approach (**Figure 1**). Moreover, CDR3s include the junction between the V gene segment and the J gene segment, and part of the D segment in heavy chains. These segments include the nucleotide deletion and insertions, that can increase the CDR3 sequence diversity beyond the pre-encoded V-D-J germline sequences. All of this makes the CDR3 the most variable region in Ig genes, and it is most often critical for antigen binding. However, its variable nature makes it hard to analyze and thus it is often excluded from the selection analysis (16). Here we propose a method that also accounts for the mutations in the CDR3, and show that CDR3 mutations have a significant impact on the results of selection analysis (**Figure 2**).

We used IgTreeZ to show that tree-based mutation analysis reveals different mutation profiles of gut B cell sub-populations. We found that naïve trees are not subject to positive nor negative selection in the CDRs, and that naïve trees’ FWRs seem to be subject to selection for replacement mutation. The latter finding is surprising, as naïve B cells are defined as cells that have not yet been exposed to an antigen, and did not undergo affinity maturation, hence their IgV genes are not expected to contain any mutations. Indeed, 60-70% of the naïve cell sequences contained no mutations, and those that did had only very few mutations per sequence (Tejedor Vaquero S et al. manuscript in preparation). There are several possible explanations for the observation of SHM in cells that were identified as naïve B cells. First, cannot completely exclude PCR errors, however these should have been eliminated by the use of unique molecular identifiers; the relatively high percentage of naïve B cell sequences with mutations also argues against this possibility. The latter consideration, and the low SHM frequency in these sequences, also argue against many of these cells having been contaminating IGM memory cells. Recent studies show that “naïve B cells” (typically IgD^high^IgM^+^CD10^-^CD27^-^CD38^low^) are more heterogeneous than expected and may include a fraction recently activated by antigen. So our naïve cells with IgV gene mutations may represent, at least in part, recently activated naïve cells which have just been instructed to become GC B cells and begun to express activation-induced cytidine deaminase (AID). An exciting possibility is that these are similar to the “activated naïve” B cells observed in systemic lupus erythematosus (7, 30) and COVID-19 (31). Whatever the explanation, the selection for replacement mutations in the FWRs is inconsistent with selection for receptor stability. Perhaps the Ig undergoes many mutations in the FWRs during early activation, with selection not yet operative, so that these results only reflect mutations and not selection. The differentiation pathways of this fraction of naïve B cells are still being elucidated; they may or may not enter the GC at a later time point, and may even participate in a wholly extrafollicular activation pathway.

We also analyzed COVID-19 patient repertoires and found indications for extensive SHM between the second and fourth week after onset of clinical symptoms and evidence for the generation of affinity maturated, structurally stable antibodies by day ~32 post-infection in the patients with mild and moderate responses. A tree-based mutation analysis of the lineage trees of all COVID-19 patients revealed a tendency of SHM to avoid mutating away from positive amino acids, but a high tendency to create them. Recently, Khan and colleagues found that an acidic tandem repeat in the Nsp3 subdomain of the HCoV-HKU1 polyprotein was the predominant target of antibody responses in adult donors (32). This may explain the positive tendency we recognized. In a review from 2021 (33), the authors note that high neutralizing antibody titers are associated with potentially extrafollicular B cell responses, and suggest that the development of neutralizing antibodies against SARS-CoV-2 can be accomplished by many B cells with little or no affinity maturation required. This is consistent with our finding that the mild disease patient shows the lowest selection pressure on the second day from the appearance of clinical symptoms.

COVID-19 patient clones that were sampled at all three time points had higher selection for replacement mutations in the CDRs (**Figures 4I, J**). Although clones shared between all three time points tend to be larger and include more mutations, the overall mutation counts had less impact on the selection than mutation location [**Figure 4B**, and as shown by (15)]. For this reason, this higher selection for replacement mutations may be part of the effort exerted by the immune system to generate efficient antibodies against the virus, an effort which may have been less effective in the patient who suffered from a severe disease.

Finally, we have shown that the output from IgTreeZ can be used in machine learning models, for example to distinguish between lymphoma and normal lineage trees. Mutation-based machine learning was recently shown to predict a sequence’s cell type (34). An accurate tree-based mutation profiling can be useful for machine learning based classification as well.

IgTreeZ is designed for repertoires. The program can process a large number of trees using parallel processing, and by default uses the maximal number of processors available, but this can be adjusted using a parameter. Using 4 CPU cores, the mutation analysis, the most complex of IgTreeZ functions, takes less than 30 minutes for 90,000 trees containing 2,000,000 sequences. Utilizing 64 CPU cores decreases the running time of the same tree set to 12 minutes. To overcome the operating system’s maximum argument number limit, IgTreeZ can receive its input trees as a directory name, and process the whole directory’s content. A tree with more than 5000 nodes is analyzed in less than two minutes, even on an 8G RAM. These features theoretically allow IgTreeZ to process any number of trees, even large ones.

In summary, B cell lineage tree analysis may shed light on many of aspects of B cell affinity maturation in GCs in particular, and on B cell response population dynamics in general. Our new tool, IgTreeZ, performs various types of lineage tree-based analysis in a simple command line mode, making all these analyses easily accessible to non-bioinformaticians. An important conclusion from our analysis is that CDR3 regions, which are often excluded from selection analyses, must be included for the results to be correct; while CDR3 inclusion is only possible if these analyses are lineage tree-based.

## Data Availability Statement

The program IgTreeZ can be found in GitHub - https://github.com/neumanh/IgTreeZ. The DLBCL data can be found in the NCBI Sequence Read Archive under accession number PRJNA796500 - https://www.ncbi.nlm.nih.gov/bioproject/PRJNA796500. The COVID-19 data were downloaded from iReceptor (Montague et al., 2021) - https://gateway.ireceptor.org/samples?query_id=55507. The healthy ileum data can be found in the NCBI Sequence Read Archive under accession number PRJNA596067, and will be released upon publication of Tejedor Vaquero S et al. (in preparation at the time of this manuscript’s publication) - https://dataview.ncbi.nlm.nih.gov/object/PRJNA596067?reviewer=fh0r7gajkn50dcpiarrdss18d9.

## Ethics Statement

The use of ileum tissue samples was approved by the Ethical Committee for Clinical Investigation of the Institut Hospital del Mar d’ Investigacions Mèdiques (CEIC-IMIM 20114/5892/I) and by the Mount Sinai Institutional Review Board (HS#14-00174). The DLBCL study was approved by the Sheba Medical Center and Israeli Ministry of Health review boards. The patients/participants provided their written informed consent to participate in this study.

## Author Contributions

HN wrote the IgTreeZ program and all other scripts described here unless otherwise noted, performed the research and wrote the manuscript. JA and HN wrote the ML scripts, performed the ML analysis, and wrote the manuscript. MK, GM, and AC contributed experimental data and wrote the manuscript. RM designed and supervised the research and wrote the manuscript. All authors contributed to the article and approved the submitted version.

## Funding

HN was supported by a Bar-Ilan University President’s Scholarship. AC is supported by the Spanish Ministry of Economy and Competitiveness (MINECO) grant RTI2018-093894-B-100. GM is supported by Spanish Institute of Health Carlos III (Miguel Servet grant 2020-2024).

## Conflict of Interest

The authors declare that the research was conducted in the absence of any commercial or financial relationships that could be construed as a potential conflict of interest.

## Publisher’s Note

All claims expressed in this article are solely those of the authors and do not necessarily represent those of their affiliated organizations, or those of the publisher, the editors and the reviewers. Any product that may be evaluated in this article, or claim that may be made by its manufacturer, is not guaranteed or endorsed by the publisher.
