# Supplementary material for: IgTreeZ, A Toolkit for Immunoglobulin Gene Lineage Tree-Based Analysis, Reveals CDR3s Are Crucial for Selection Analysis
Source: Front Immunol. 2022 Oct 26;13:822834. doi: 10.3389/fimmu.2022.822834 (PMC9643157; doi:10.3389/fimmu.2022.822834)
Supplement: Supplementary file 1 [file DataSheet_1.docx]

Supplementary Material

#
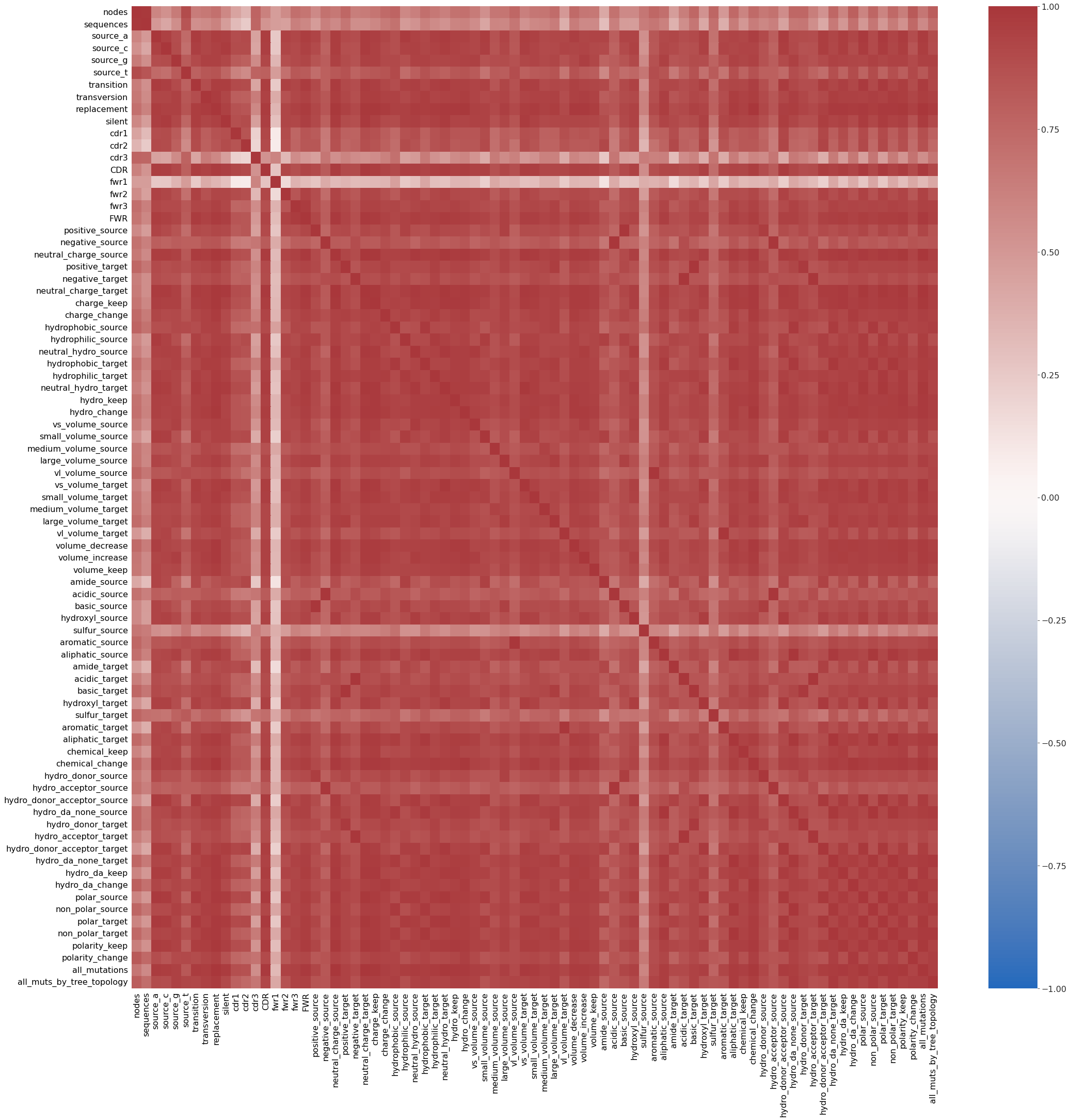
Supplementary Figures

**Supplementary Figure S1.** **Correlation matrix of** **IgTreeZ-mutation analysis results.** The matrix represents the correlations between the features defined in Supplementary Table 1.

#
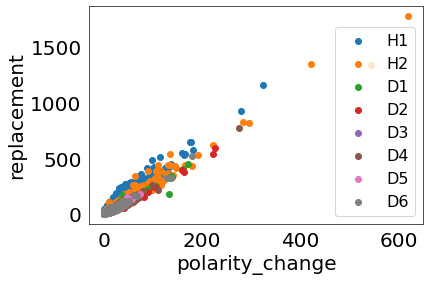

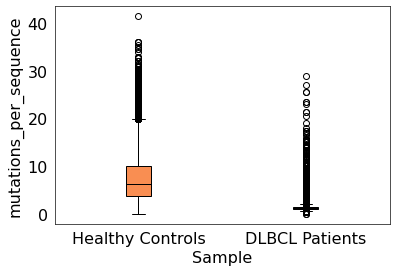

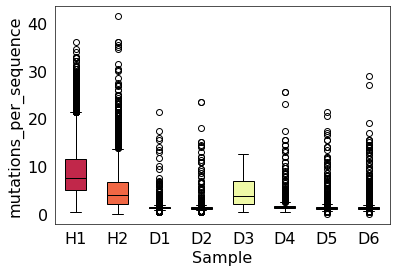

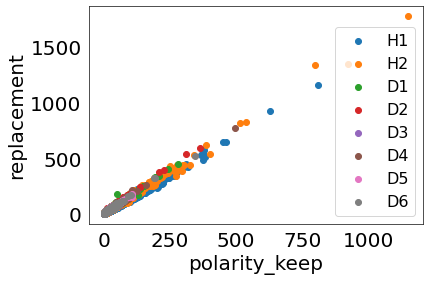


**B**

**A**

**C**

**D**

**Supplementary Figure S2.** **Feature inspection**. **A,B.** Mutations per sequence distribution per sample **(A)** and per group **(B)**. **C.** Relationship between the numbers of replacement mutations that retain amino-acid polarity and the total numbers of mutations, tallied per tree. **D.** Relationship between the numbers of replacement mutations that change amino-acid polarity and total numbers of mutations, tallied per tree.

**A**

**B**


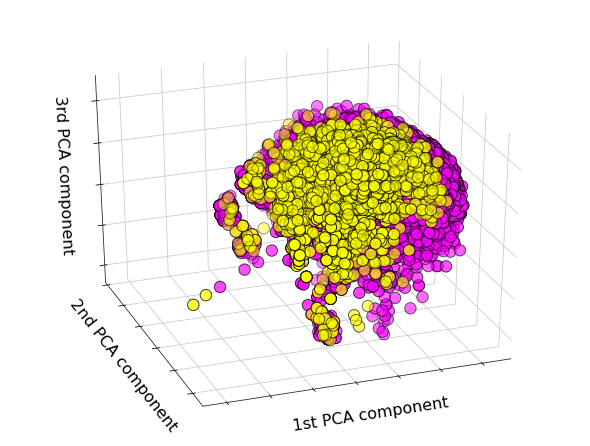
**
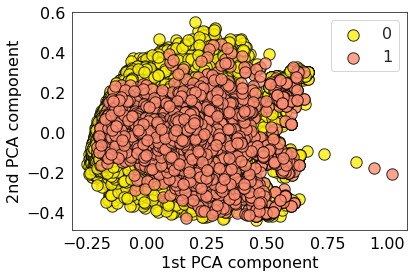
**

**C**

**
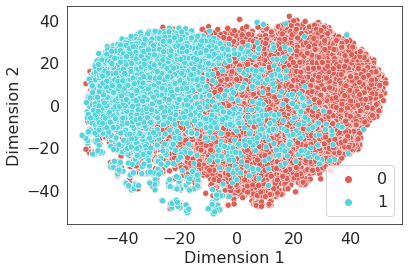
**

**Supplementary Figure S3. Dimensionality Reduction of IgTreeZ-mutation analysis results. A.** two-dimensional PCA. **B.** three-dimensional PCA. **C.** T-SNE

**A**


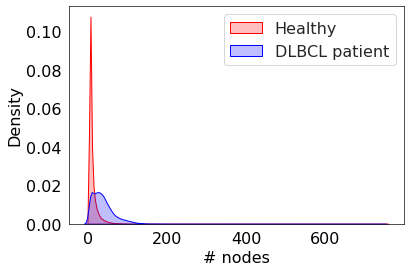

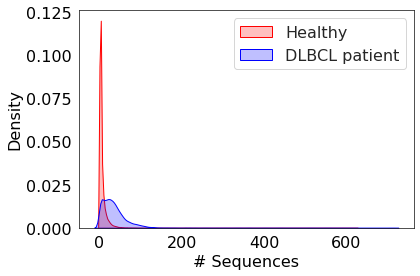


**C**

**B**


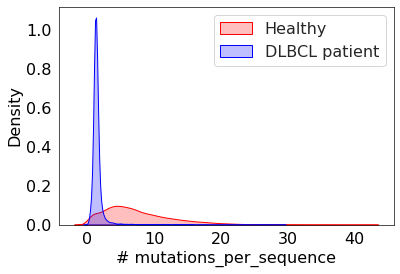


**Supplementary Figure S4. Distribution of the most important features identified by the Random Forest and the AdaBoost models. A.** The number of sequences in each tree, as counted by IgTreeZ-mutations. **B.** The number of nodes in each tree, as counted by IgTreeZ-mutations. **C.** The number of mutations per sequence in each tree.

# Supplementary Tables

**Supplementary Table 1: Feature descriptions**

| **Name** | **Description** |
| --- | --- |
| sample | Sample name, as given by the user |
| Tree ID | A numerical tree ID, taken from the file name or tree name (in AIRR scheme file) |
| nodes | Number of nodes in a tree |
| sequences | Number of observed nodes in a tree |
| source_a | Number of mutations from adenosine |
| source_c | Number of mutations from cytidine |
| source_g | Number of mutations from guanosine |
| source_t | Number of mutations from thymidine |
| transition | Number of mutations that involve transition |
| transversion | Number of mutations that involve transversion |
| replacement | Number of replacement mutation |
| silent | Number of silent mutations |
| cdr1 | Number of mutations in the CDR1 region |
| cdr2 | Number of mutations in the CDR2 region |
| cdr3 | Number of mutations in the CDR3 region |
| CDR | Number of mutations in all CDRs |
| fwr1 | Number of mutations in the FWR1 region |
| fwr2 | Number of mutations in the FWR2 region |
| fwr3 | Number of mutations in the FWR3 region |
| FWR | Number of mutations in all FWRs |
| positive_source | Number of mutations that start in positive AAs |
| negative_source | Number of mutations that start in negative AAs |
| neutral_charge_source | Number of mutations that start in a neutral charge AAs |
| positive_target | Number of mutations into positive AAs |
| negative_target | Number of mutations into negative AAs |
| neutral_charge_target | Number of mutations into neutral charge AAs |
| charge_keep | Number of REPLACEMENT mutations that keep the same charge |
| charge_change | Number of mutations that change the charge |
| hydrophobic_source | Number of mutations that start in hydrophobic AAs |
| hydrophilic_source | Number of mutations that start in hydrophilic AAs |
| neutral_hydro_source | Number of mutations that start in neutral-hydropathy AAs |
| hydrophobic_target | Number of mutations into hydrophobic AAs |
| hydrophilic_target | Number of mutations into hydrophilic AAs |
| neutral_hydro_target | Number of mutations into neutral-hydropathy AAs |
| hydro_keep | Number of REPLACEMENT mutations that keep the same hydropathy |
| hydro_change | Number of mutations that change the  hydropathy |
| vs_volume_source | Number of mutations that start in very small volume AAs |
| small_volume_source | Number of mutations that start in small volume AAs |
| medium_volume_source | Number of mutations that start in medium volume AAs |
| large_volume_source | Number of mutations that start in large volume AAs |
| vl_volume_source | Number of mutations that start in very large volume AAs |
| vs_volume_target | Number of mutations into very small volume AAs |
| small_volume_target | Number of mutations into small volume AAs |
| medium_volume_target | Number of mutations into medium volume AAs |
| large_volume_target | Number of mutations into large volume AAs |
| vl_volume_target | Number of mutations into very large volume AAs |
| volume_decrease | Number of mutations that decrease the AA volume |
| volume_increase | Number of mutations that increase the AA volume |
| volume_keep | Number of REPLACEMENT mutations that keep the AA volume |
| amide_source | Number of mutations that start in amide AAs |
| acidic_source | Number of mutations that start in acidic AAs |
| basic_source | Number of mutations that start in basic AAs |
| hydroxyl_source | Number of mutations that start in hydroxyl AAs |
| sulfur_source | Number of mutations that start in sulfur AAs |
| aromatic_source | Number of mutations that start in aromatic AAs |
| aliphatic_source | Number of mutations that start in aliphatic AAs |
| amide_target | Number of mutations into amide AAs |
| acidic_target | Number of mutations into acidic AAs |
| basic_target | Number of mutations into basic AAs |
| hydroxyl_target | Number of mutations into hydroxyl AAs |
| sulfur_target | Number of mutations into sulfur AAs |
| aromatic_target | Number of mutations into aromatic AAs |
| aliphatic_target | Number of mutations into aliphatic AAs |
| chemical_keep | Number of REPLACEMENT mutations that keep the AA chemical properties |
| chemical_change | Number of mutations that change the AA chemical properties |
| hydro_donor_source | Number of mutations that start in hydrogen donor AAs |
| hydro_acceptor_source | Number of mutations that start in hydrogen acceptor AAs |
| hydro_donor_acceptor_source | Number of mutations that start in hydrogen donor and acceptor AAs |
| hydro_da_none_source | Number of mutations that start in AAs that have no hydrogen donor or acceptor atoms |
| hydro_donor_target | Number of mutations into hydrogen donor AAs |
| hydro_acceptor_target | Number of mutations into hydrogen acceptor AAs |
| hydro_donor_acceptor_target | Number of mutations into hydrogen donor and acceptor AAs |
| hydro_da_none_target | Number of mutations into AAs that have no hydrogen donor or acceptor atoms |
| hydro_da_keep | Number of REPLACEMENT mutations that keep the AA hydrogen-donor-acceptor tendency |
| hydro_da_change | Number of mutations that change the AA hydrogen-donor-acceptor tendency |
| polar_source | Number of mutations that start in polar AAs |
| non_polar_source | Number of mutations that start in non-polar AAs |
| polar_target | Number of mutations into polar AAs |
| non_polar_target | Number of mutations into non-polar AAs |
| polarity_keep | Number of REPLACEMENT mutations that keep the AA polarity |
| polarity_change | Number of mutations that change the AA polarity |
| all_mutations_but_fwr1 | All the mutations in FWR2, FWR3, CDR1, CDR2 and CDR3 region |
| mutations_per_sequence | The number of all_mutations_but_fwr1 divided by the number of sequences |

**Supplementary Table 2: Parameters and performance summary of the KNN, Decision Trees, Random Forest, AdaBoost and LDA models**

| **Algorithm** | **Parameter definition** | **Parameter grid values** | **Optimal hyperparameter values** | | **Macro avg** |
| --- | --- | --- | --- | --- | --- |
| KNN | **n_neighbors-** number of neighbours. | [2,4,10] | [4] | | **Training set**:  0.94642  **Test set:**  0.94427 |
|  | **weights-** weight function used in prediction. | ['uniform', 'distance'] | ['uniform'] | |  |
|  | **leaf_size-** the leaf size that is passed to BallTree or KDTree and can affect the speed as well as the memory. | [20, 30,40,50] | [20] | |  |
|  | **p-** the power parameter for the Minkowski metric. | [1,2] | | [1] |  |
| Decision Trees | **criterion**- the function to measure the quality of a split. | ['entropy', 'gini'] | [gini] | | **Training set**:  0.94868  **Test set:**  0.93639 |
|  | **splitter**- the strategy used to choose the split at each node. | ['best', 'random'] | [random] | |  |
|  | **max_depth**- the maximum depth of the tree (if None, then nodes are expanded until all leaves contain less than min_samples_split samples). | [None, 10, 20, 40, 80] | [10] | |  |
|  | **min_samples_split**- the minimum number of samples required to split an internal node. | [1, 2, 8, 16] | [2] | |  |
|  | **min_samples_leaf-** The minimum number of data points allowed in a leaf node. | [1, 2, 4] | | [4] |  |
| Random Forest | **n_estimators**- the number of trees in the forest. | [10, 50, 100] | [50] | | **Training set**:  0.98026  **Test set:**  0.95170 |
|  | **max_features**- the number of features to consider when looking for the optimal split. | ['auto', 'sqrt', 'log2'] | [log2] | |  |
|  | **min_sample_split**- the minimum number of samples required to split an internal node. | [1, 2, 8, 16] | [8] | |  |
|  | **criterion**- the function to measure the quality of a split. | ['entropy', 'gini'] | | [gini] |  |
| AdaBoost | **n_estimators**- the maximum number of estimators at which boosting is terminated. | [20, 50, 100, 200] | [200] | | **Training set**:  0.94406  **Test set:**  0.94014 |
|  | **learning_rate-** the weight applied to each classifier at each boosting iteration. | [0.5, 1, 2, 4] | [1] | |  |
|  | **algorithm-** whether to use the SAMME.R real boosting algorithm or the SAMME discrete boosting algorithm | ['SAMME', 'SAMME.R'] | | ['SAMME.R'] |  |
| LDA | **solver-** a solver to use singular value decomposition, least-squares solution or eigenvalue decomposition. | ['svd', 'lsqr', 'eigen'] | ['svd'] | | **Training set**: 0.94426  **Test set:**  0.95023 |
|  | **shrinkage-** the shrinkage parameter - no shrinkage or automatic shrinkage using the Ledoit-Wolf lemma. | ['auto', None] | [None] | |  |

**Supplementary Table 3: Top 5 most important features by the Random Forest model**

|  | **Features** | **Importance** |
| --- | --- | --- |
| **Variable 0** | sequences | 0.19 |
| **Variable 1** | nodes | 0.14 |
| **Variable 2** | cdr3 | 0.07 |
| **Variable 3** | all_mutations | 0.04 |
| **Variable 4** | source_g | 0.03 |

**Supplementary Table 4: Top 5 most important features by the AdaBoost model**

|  | **Features** | **Importance** |
| --- | --- | --- |
| **Variable 0** | sequences | 0.08 |
| **Variable 1** | mutations_per_sequence | 0.08 |
| **Variable 2** | CDR | 0.06 |
| **Variable 3** | FWR | 0.06 |
| **Variable 4** | all_mutations | 0.06 |
